# Isolation and Characterization of vB_kpnM_17-11, a Novel Phage Efficient Against Carbapenem-Resistant *Klebsiella pneumoniae*

**DOI:** 10.3389/fcimb.2022.897531

**Published:** 2022-07-05

**Authors:** Jiawei Bai, Feiyang Zhang, Shuang Liang, Qiao Chen, Wei Wang, Ying Wang, Alberto J. Martín-Rodríguez, Åsa Sjöling, Renjing Hu, Yingshun Zhou

**Affiliations:** ^1^ Department of Pathogen Biology, School of Basic Medicine, Public Center Experimental Technology of Pathogen Biology, Southwest Medical University, Luzhou, China; ^2^ Department of Microbiology, Tumor and Cell Biology, Karolinska Institutet, Solna, Sweden; ^3^ Department of Laboratory Medicine, The Affiliated Wuxi No.2 People’s Hospital of Nanjing Medical University, Wuxi, China

**Keywords:** phage, *Klebsiella pneumonia*, depolymerase, phage therapy, animal model

## Abstract

Phages and phage-encoded proteins exhibit promising prospects in the treatment of Carbapenem-Resistant *Klebsiella pneumoniae* (CRKP) infections. In this study, a novel *Klebsiella pneumoniae* phage vB_kpnM_17-11 was isolated and identified by using a CRKP host. vB_kpnM_17-11 has an icosahedral head and a retractable tail. The latent and exponential phases were 30 and 60 minutes, respectively; the burst size was 31.7 PFU/cell and the optimal MOI was 0.001. vB_kpnM_17-11 remained stable in a wide range of pH (4-8) and temperature (4-40°C). The genome of vB_kpnM_17-11 is 165,894 bp, double-stranded DNA (dsDNA), containing 275 Open Reading Frames (ORFs). It belongs to the family of *Myoviridae*, order Caudovirales, and has a close evolutionary relationship with *Klebsiella* phage PKO111. Sequence analysis showed that the 4530 bp *orf022* of vB_kpnM_17-11 encodes a putative depolymerase. *In vitro* testing demonstrated that vB_kpnM_17-11 can decrease the number of *K. pneumoniae* by 10^5^-fold. In a mouse model of infection, phage administration improved survival and reduced the number of *K. pneumoniae* in the abdominal cavity by 10^4^-fold. In conclusion, vB_kpnM_17-11 showed excellent *in vitro* and *in vivo* performance against *K. pneumoniae* infection and constitutes a promising candidate for the development of phage therapy against CRKP.

## Introduction

*K. pneumoniae* is an opportunistic pathogen which often causes pneumonia, pyogenic liver abscess (PLA), necrotizing fasciitis and urinary tract infections (UTIs) (Fu et al., 2018). In recent years, multidrug-resistant bacteria especially Carbapenem-Resistant *Klebsiella pneumoniae* (CRKP), had posed a huge challenge to public health, with the emergence of a growing number of CRKP isolates worldwide (Zhang et al., 2021b; Zhang et al., 2021a). Thus, it is an urgent need to find novel ways to prevent and control CRKP infections.

As a potential antibacterial agent, phages can effectively and specifically infect host bacteria (Onsea et al., 2021). Nonetheless, phages often have distinct biological characteristics such as host range, proliferation rates, temperature and pH stability, which altogether limit the industrial production of phage preparations (Cai et al., 2019). Therefore, the isolation and characterization of phages with appropriate characteristics is of great significance.

In addition to their biological properties, advancing knowledge on phage genomics has intrinsic fundamental importance. By exploring the phage genome, we can analyze the genetic characteristics of phages and develop new phage-based antibacterial agents. Researchers often modularize phage genes to analyze the differences between the same functional modules, it is helpful to explore genetic and functional similarities in different phage genomes (Zhou et al., 2018). Phage genes can mutate during the proliferation process. The mutation rate of the individual phage genes differ depending on the evolutionary pressures they are subjected to (Comeau et al., 2007). For example, the gene encoding the phage head structure has a low probability to mutate. As a conserved protein, the phage major capsid protein (MCP) is often used to reconstruct the evolutionary relationships of phages (Zhao et al., 2019). In addition, phage tail genes coding products may have antibacterial potential, such as the depolymerase (Chen et al., 2020). The phage depolymerase is a protein that can specifically degrade bacterial surface capsular polysaccharides (CPS). While degrading CPS, the depolymerase simultaneously pulls the phage particles closer to the host surface and eventually binds to the outer membrane receptor (Michaud et al., 2003). Recent studies have shown that phage depolymerase can specifically recognize bacterial capsular types, remove bacterial biofilms, and effectively reduce bacterial virulence. Therapies based on phages and phage depolymerase brings new hope for the treatment of drug-resistant bacterial infections.

In previous studies, phage therapy was effectively employed in the treatment of prosthetic joint infection caused by *Staphylococcus aureus* (Ferry et al., 2020). In the context of COVID-19, phage therapy was successfully applied on secondary *Acinetobacter baumannii* infection in COVID-19 patients (Wu et al., 2021). However, the clinical application of phages has been limited due to a number of shortcomings including the narrow host range of phages or the development of resistance against phage infection by bacteria, among other factors (Labrie et al., 2010). Therefore, the isolation of new phages and the characterization of their application prospects for the treatment of pernicious infections is currently a preclinical research need. With these premises, in this study we set to isolate new phages efficient against the treatment of CRKP, identify the main genomic characteristics through genome sequencing and bioinformatic analysis, and explore their therapeutic efficacy in a mouse model of infection.

## Materials and Methods

### Bacterial Strain, Phage Isolation and Identification

*K. pneumoniae* 17-11 carrying *bla*_KPC-2_ (WGS accession: KZ984141) was used as a host for phage isolation. *K. pneumoniae* 17-11 is resistant to an array of antibiotics (**Supplementary Table 1**). A wastewater sample was collected from Affiliated Hospital of Southwest Medical University in Luzhou, Sichuan Province, China. One mL exponential phase *K. pneumoniae* 17-11 and 10 mL Luria-Bertani (LB) medium were added into 100 mL wastewater sample. This mixture was incubated at room temperature overnight, followed by centrifugation at 12,000 rpm for 10 min. The supernatant was filtered through a 0.22 μm filter to obtain acrude phage solution, which was subsequently serially diluted ten-fold from 10^-1^ to 10^-5^. One-hundred μL of phage crude solution of each dilution gradient was mixed with an equal volume of logarithmic phase 17-11 solution, and incubated at room temperature for 15 min. The mixture was added to 4 mL semi-solid medium (agar 0.5%), poured over an LB broth agar plate, and cultured overnight at 37°C (double-layer plate method). Single phage plaques were picked with an inoculation needle and inoculated into a logarithmic phase 17-11 bacterial solution. When the solution was clear, indicating full bacterial lysis, it was centrifuged and filtered, and the phage titer was detected by the double-layer plate method. The procedure above was repeated 5 times (Kropinski et al., 2009). The phage solution was dripped onto a copper mesh and stained with 2% phosphotungstic acid (PTA). An HT 7800 transmission electron microscope was used to image virions at 80 kV (Hitachi, Tokyo, Japan) in FuXin (Shanghai, China) to analyze the morphological features of individual phage particles.

### Optimal Multiplicity of Infection and One-Step Growth Curve Estimation

Five-hundred μL of a bacterial solution (10^6^ CFU/mL) were mixed with phages (10^8^, 10^7^, 10^6^, 10^5^, 10^4^, 10^3^, or 10^2^ PFU/mL) and incubated at room temperature for 15 min. Nine mL of LB medium was added to each group, and cultured at 37°C, 220 rpm, for 2 h. The mixture was centrifuged at 8,000 rpm for 10 min at 4°C, leaving the supernatant. The double-layer plate method was used to determine the phage titers of the mixture and the highest production was defined as the optimal MOI.

The one-step growth curve determination method was improved based on a previous study (Peng and Yuan, 2018). Phages and *K. pneumoniae* 17-11 were mixed at the optimal MOI and incubated for 10 min at 37°C. The mixture was then centrifuged at 4°C, 12,000 rpm for 2 min to remove free phages. The sediment was resuspended in 10 mL of LB broth. Aliquots (100 μL) were taken every 10 min from 0 min to 120 min to determine the phage titers. The burst size was defined as the number of phage progeny/number of latent infected cells.

### Host Range Evaluation, Thermal and pH Stability

The host range of phage was defined based on 92 bacterial strains. 76 strains of *K. pneumoniae*, 1 staphylococcus aureus strain, and 5 strains of *Pseudomonas aeruginosa*, *Escherichia coli* and *Salmonella*, respectively. One-hundred µL of a phage suspension containing 10^5^ PFU/mL was mixed with 100 µL 10^8^ CFU/mL bacterial solution. The double-layer plate method was used to observe the development of plaques. Samples that produced a plaque were assumed to be lysed by phages. The lysed strains were serotyped by amplification and sequencing of the CD1-VR2-CD2 region, a variable sequence coding region of the *wzc* gene (Primer: F: 5’- 3’ GGGTTTTTATCGGGTTGTAC, R: 5’-3’ TTCAGCTGGATTTGGTGG) (Pan et al., 2013).

To determine phage stability in the cold, seven mL of a phage suspension (10^8^ PFU/mL) were divided into 7 microcentrifuge tubes, and all tubes were placed in a refrigerator at 4°C. Starting from 0 min, one tube was taken out every 10 min and the phage titer was detected by the double-layer plate method. The phage thermal stability tests at 25°C, 37°C, 40°C, 50°C, 60°C and 70°C were performed in an analogous way using a water bath at the appropriate temperature.

Phage stability at different pH values was determined using Saline-Magnesium (SM) buffer, pH 1-14, in individual 15 mL centrifuge tubes. One mL of phage suspension containing 10^9^ PFU were added to each tube, incubated at room temperature for 1 h, and the phage titers counted by the double-layer plate method.

### Phage DNA Extraction, Genome Sequence, and Bioinformatic Analysis

Phage DNA was extracted using the virus DNA\RNA Extraction Kit (TaKaRa Mini BEST Viral RNA/DNA Extraction Kit Ver.5.0) according to the manufacturer’s protocol. The phage genome was sequenced at Majorbio (Shanghai, China). RAST was used to predict and annotate the ORFs. tRNAscan-SE (Chan and Lowe, 2019) was used to analyze viral tRNA genes. BLAST Ring Image Generator v0.95 (BRIG) (Alikhan et al., 2011) was used to map the whole genome and analyze the distribution of phage genes. BLAST+ (version 2.11.0) and Artemis Comparison Tool (ACT, version 18.1.0) (Carver et al., 2005) were used to carry out multiple genome comparisons to study differences in gene arrangement between phages. Phylogenies were reconstructed with MEGA-X by the Neighbor-Joining method with 1000 bootstrap replicates (Kumar et al., 2018). HHpred (Söding et al., 2005) software was used to predict depolymerase.

### Bactericidal Effect of the Phage *in Vitro* and *in Vivo*

To determine the bactericidal effect of the phage *in vitro*, *K. pneumoniae* 17-11 was cultured to exponential phase in 150 mL LB medium. The bacterial culture was then divided into 3 conical bottles. Add 1 mL Phosphate Buffer Solution (PBS), 1 mL phage, 1 mL polymycin B (PB, Final concentration 2 μg/mL) to three conical bottles, respectively (Li et al., 2020). Samples (200 µL) were taken every 30 min for a total of 150 min, and the OD_595_ was measured at each time point.

Two experiments were designed to investigate the bacteriostatic effect of the phage *in vivo*. First, 20 mice were given cyclophosphamide (CTX) 300 mg/kg for 3 days in advance to construct the immunodeficient mouse models (Manepalli et al., 2013). Ten immunodeficient mice were injected with 500 μL PBS containing 10^7^ CFU *K. pneumoniae* 17-11 and randomly divided into two groups: one group was treated with 500 μL of a phage suspension of 10^9^ PFU/mL, and the other group was injected with 500 μL PBS as a control. The survival curves of the mice were recorded every 12 h up to 148 h. Other 10 immunodeficient mice were injected with 500 μL PBS containing 5 × 10^6^ CFU *K. pneumoniae* 17-11 and also randomly divided into two groups: one group was treated with 500 μL phage suspension (10^9^ PFU/mL) and the other group was injected with 500 μL PBS. Ten mice were sacrificed by cervical dislocation at 24 h post infection and peritoneal lavages were collected with 1 mL of sterile PBS, then plated on LB plates for colony enumeration. A summary of the *in vivo* experimental design is shown in **Supplementary Figure 1**. All experiments were conducted in compliance with approved protocols and guidelines of the Southwest Medical University Animal Care and Use Committee (20211124-002).

### Statistical Analysis

All analyses were performed using Prism 8 software (GraphPad Software, CA, USA). All experimental data were expressed as mean ± SD. Differences in survival curves were tested with the Gehan-Breslow-Wilcoxon test. The two groups were compared using the Mann–Whitney test. *P* < 0.05 was deemed to be statistically significant.

## Results

### Isolation and Morphology of Phage

**Figure 1:**
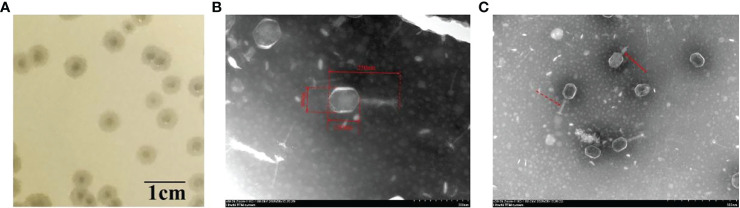
Plaques and transmission electron microscopy of phage vB_kpnM_17-11. **(A)** Plaque morphology of phage vB_kpnM_17-11 on a bacterial lawn of *K. pneumoniae* 17-11 in nutrient broth with 0.5% agar (scale bar = 1 cm). **(B)** Transmission electron microscopy of phage vB_kpnM_17-11. **(C)** Contractive and non-contractive tails. Dashed arrow for non-contractile tail; Solid arrow for contractile tail.

A novel *K. pneumoniae* phage was isolated and named it vB_kpnM_17-11. vB_kpnM_17-11 can form transparent plaques of 3 mm in diameter on a double agar plate. The halo surrounding the plaque indicates that the phage has depolymerase activity (**Figure 1A**). Transmission electron microscopy showed that the length of vB_kpnM_17-11 was 250 nm (**Figure 1B**). The phage capsid is an icosahedron with a length and width of 120 nm and 90 nm, respectively. The retractable tail structure of vB_kpnM_17-11 is a typical feature of the *Myoviridae* phage family (**Figure 1C**).

### The MOI and One-Step Growth Curve of vB_kpnM_17-11

To determine the optimal MOI, suspensions of vB_kpnM_17-11 phage particles containing 10^2^-10^8^ PFU/mL were mixed with *K. pneumoniae* 11-17 cells (10^6^ CFU/mL). The optimal MOI giving the highest titer of progeny phages was determined to be 0.001 (**Table 1**).

**Table 1:** Optimal MOI for phage.

MOI	Phage (PFU/mL)	Host (CFU/mL)	Product (PFU/mL)
100	10^8^	10^6^	1.41 × 10^7^
10	10^7^	10^6^	2.63 × 10^7^
1	10^6^	10^6^	1.57 × 10^8^
0.1	10^5^	10^6^	2.80 × 10^8^
0.01	10^4^	10^6^	5.05 × 10^8^
0.001	10^3^	10^6^	2.00 × 10^9^
0.0001	10^2^	10^6^	1.15 × 10^9^

Phages and bacteria were mixed according to different MOI and the titer of the progeny phages was measured.

**Figure 2:**
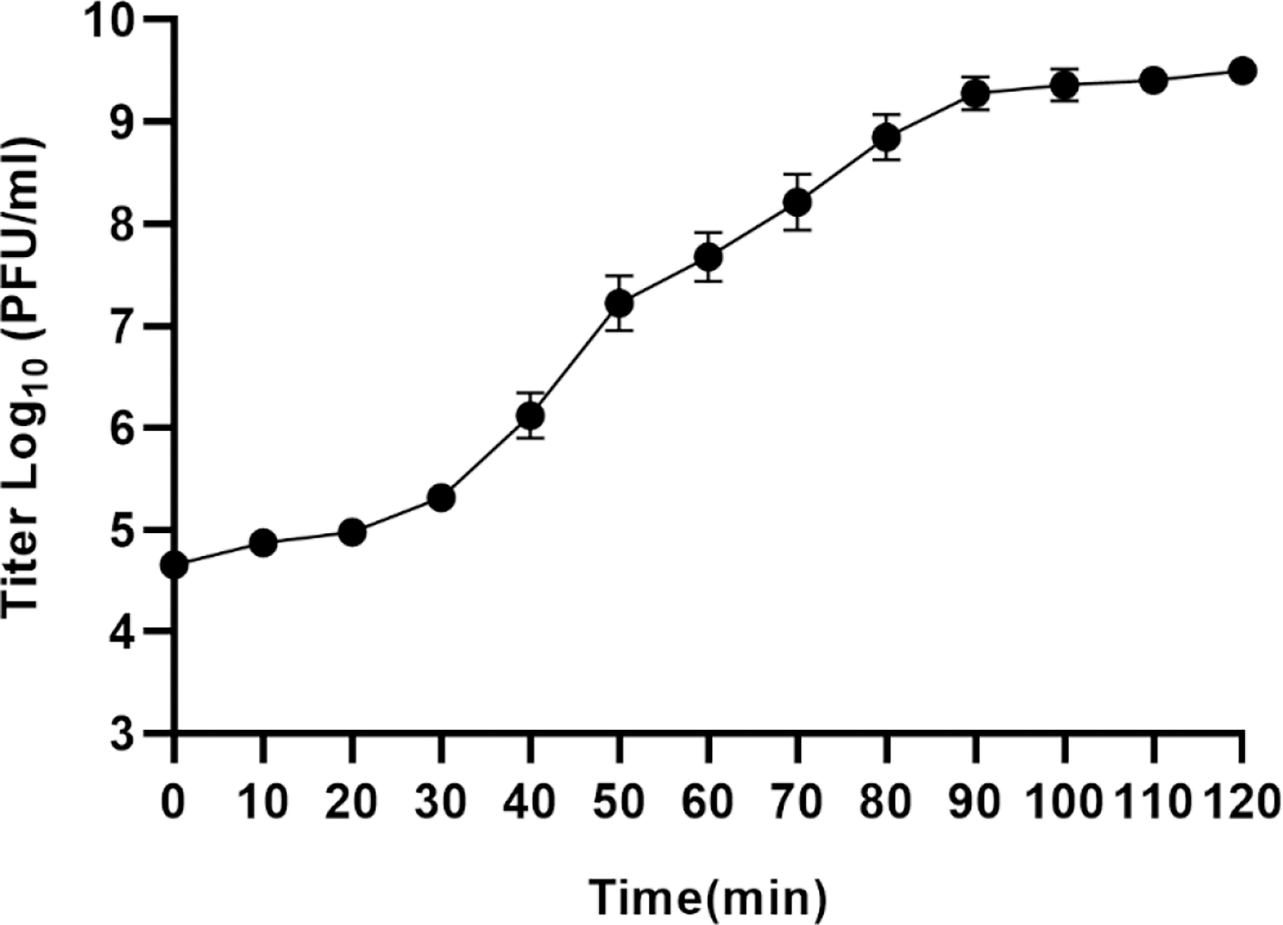
One-step growth curve of vB_kpnM_17-11. The PFU of each infected cell is shown at different time points. Each data point represents the mean of three independent experiments, and the error bar represents the standard deviation.

To analyze phage proliferation *in vitro*, a one-step growth curve was performed (**Figure 2**). From this analysis, the latent phase of vB_kpnM_17-11 was determined to be 30 min, in which phage proliferation was negligible. Between 30 min and 90 min post inoculation, phage titers increased exponentially, reaching a plateau after 90 min. The burst size was 31.7 PFU/cell after the exponential phase.

### Host Range, Thermal and pH Stability

**Figure 3:**
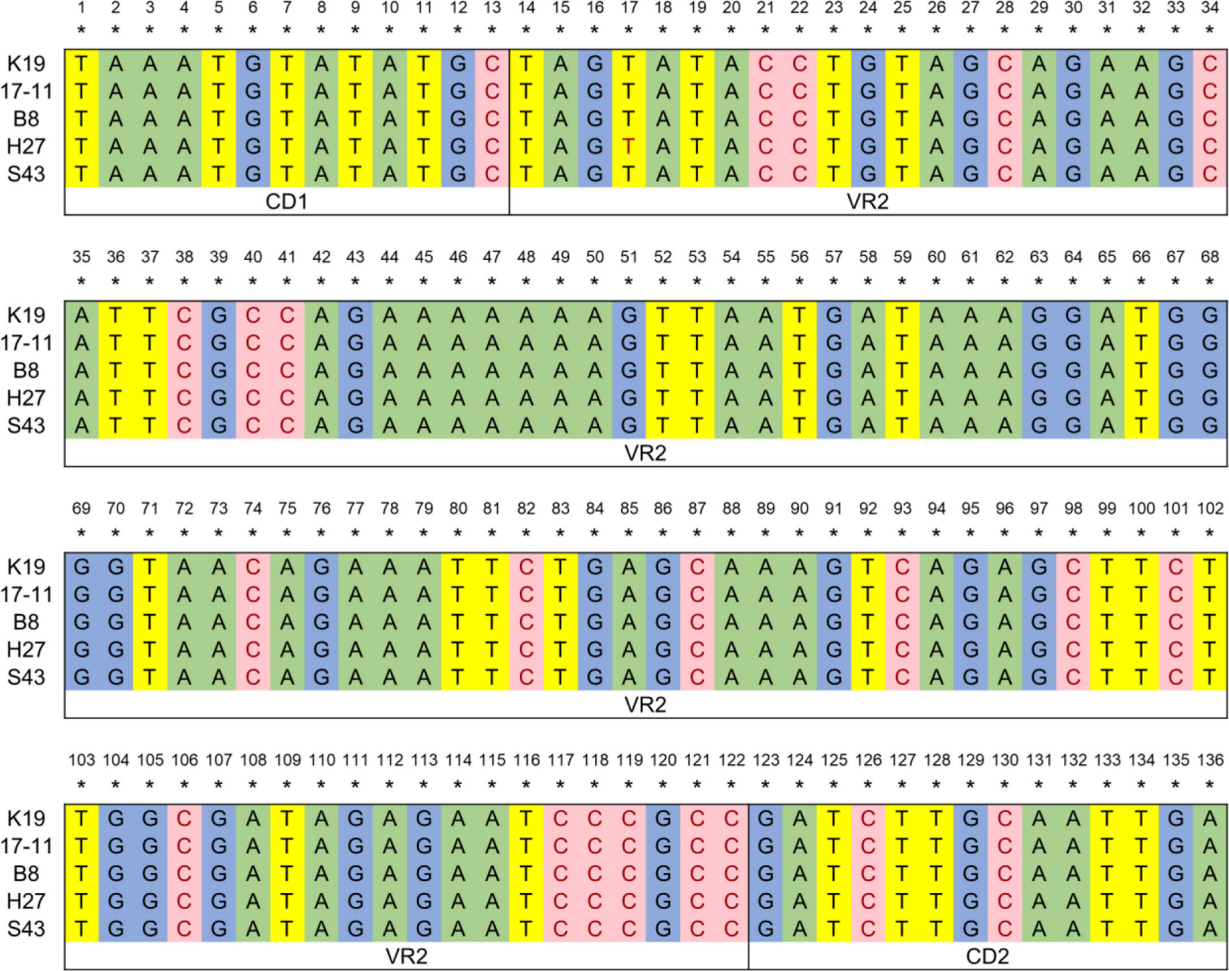
Serotype identification of *k. pneumoniae*. The CD1-VR2-CD2 region of the *wzc* gene was amplified by PCR, sequenced and compared to determine the serotype of *k. pneumoniae* lysed by vB_kpnM_17-11. K19 represents the inherent sequence of CD1-VR2-CD2 region of *wzc* gene of *k. pneumoniae* serotype K19, and 17-11, B8, H27, and S43 are four strains that can be lysed by vB_kpnM_17-11 respectively.

**Figure 4:**
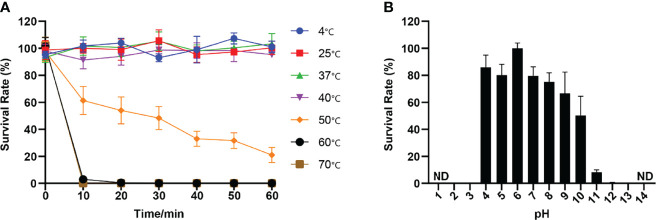
Thermal and pH stability of vB_kpnM_17-11. **(A)** Temperature stability of phages vB_kpnM_17-11. When incubated at different temperatures for 60 min, the phages could survive at 4°C, 25°C, 37°C and 40°C. At 50°C, the phage concentration decreased by 80%. Phages could not survive at 60°C and 70°C. Data were obtained from three independent experiments and are shown as mean ± standard deviation. **(B)** PH stability of phages vB_kpnM_17-11. vB_kpnM_17-11 was incubated at the indicated pH conditions for 60 min. vB_kpnM_17-11 is relatively stable under neutral condition. The phage titer decreased significantly under acidic or alkaline conditions. vB_kpnM_17-11 titer extreme acid-base conditions cannot be detected (ND, Not Detected). Data were obtained from three independent experiments and are shown as mean ± standard deviation.

vB_kpnM_17-11 was able to infect 4.35% (4/92) of the tested strains (**Supplementary Table 2**). Serotyping experiments upon sequencing of the *wzc* gene CD1-VR2-CD2 region of the 4 strains of *K. pneumoniae* lysed by vB_kpnM_17-11 demonstrated the 4 strains to belong to the K19 serotype (**Figure 3**). The phage titer remained stable at ambient temperatures from 4°C to 40°C. At 50°C, phage titers decreased by 79% after 60 min. Complete loss of viability after 10 min was recorded when the temperature was ≥ 60°C (**Figure 4A**). The titer of vB_kpnM_17-11 was relatively stable at pH 4 - 10 (> 50%). The phage titer decreased significantly in acidic (pH 2-3) or alkaline (pH 11-13) conditions. Phage titers could not be detected at pH 1 and 14 (**Figure 4B**).

### Genomic Analysis of the Phage vB_kpnM_17-11

The genome sequence of phage vB_kpnM_17-11 is available from GenBank under accession MW239157. vB_kpnM_17-11 has dsDNA with a genome size of 165,894 bp, a G + C content of 39.5%, and a gene density of 1.66 (Gene density: Gene number/1000 bp). The coding density of vB_kpnM_17-11 genome was 93.0%, therefore only 7% of the genome represented non-coding sequences. A total of 16 tRNA genes were predicted. The absence of lysogen-formation genes in the vB_kpnM_17-11 indicates that vB_kpnM_17-11 is a lytic phage.

**Figure 5:**
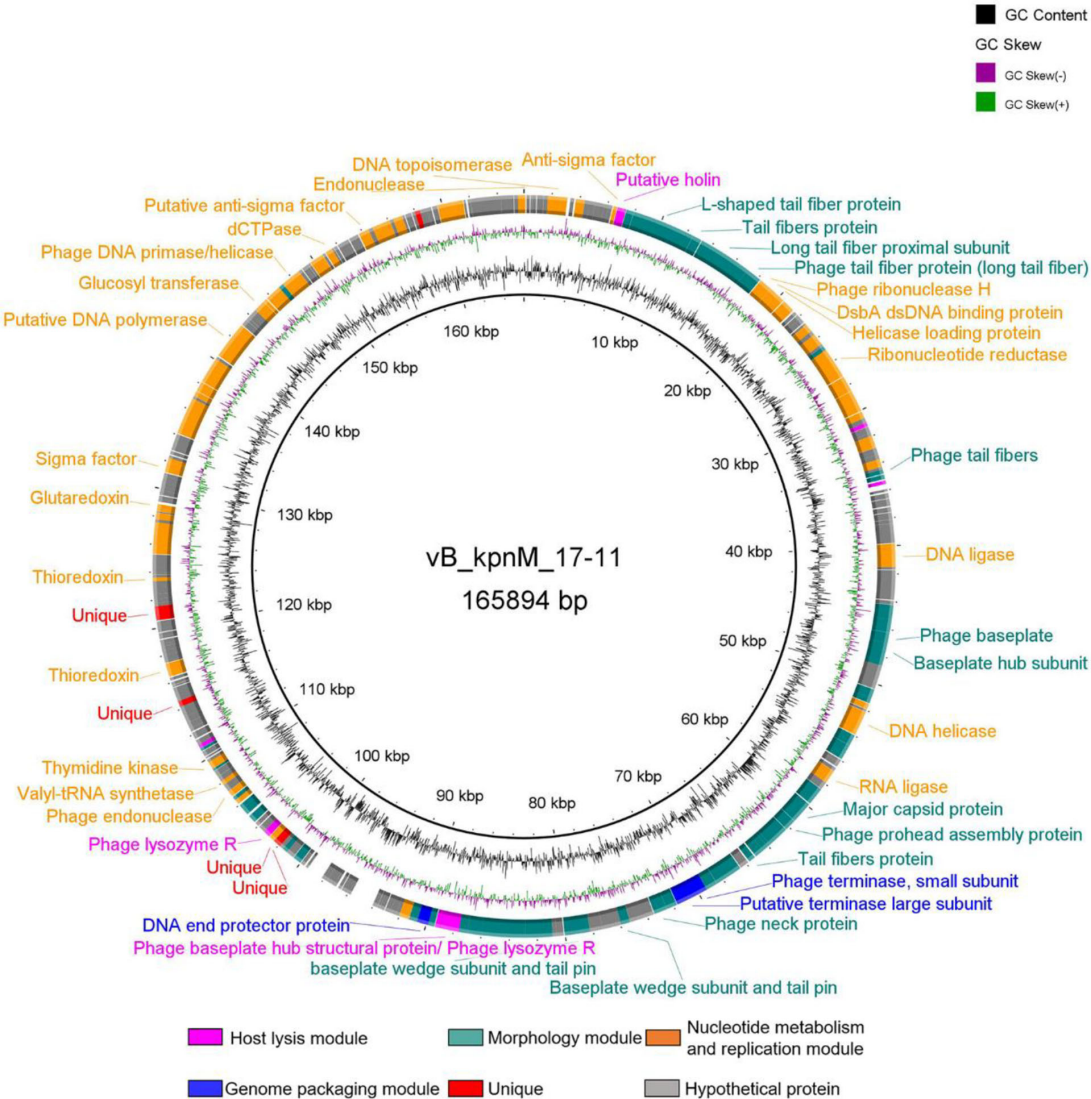
Genome map of phage vB_kpnM_17-11. The black circle in the middle represents the GC content; the violet circle represents GC skew. Different colors represent genes with different functions: peach for host lysis module, green for morphology module, orange for nucleotide metabolism and replication, blue for packaging module, red for specific protein, gray for hypothetical protein.

The vB_kpnM_17-11 genome encodes 275 ORFs (**Supplementary Table 3**) ranging from 30 to 1510 codons. 39.6% (109/275) of the ORFs encoded functional genes and were divided into four functional modules (**Figure 5**). First, 49.1% (54/109) of the functional ORFs are predicted to be involved in nucleotide metabolism and replication in association with a host, covering most processes of phage replication, regulation, transcription, and translation. Second, 41.3% (45/109) of the functional ORFs encode structural products such as phage head and tail morphology. Third, 3.67% (4/109) of the functional ORFs are predicted to be used to form packaging module. ORF96 encoded a portal vertex protein; ORF104 and ORF105 encoded a terminase; and ORF121 encoded a DNA ended protector protein. Fourth, vB_kpnM_17-11 has 5.5% (6/109) functional ORFs involved in host lysis, including a putative holin (ORF020), a Rz1 protein (ORF047), a lysis inhibition accessory protein (ORF058), a phage baseplate hub structural protein/Phage lysozyme R (ORF119), a phage lysozyme R (ORF147) and a rI lysis inhibition regulator membrane protein (ORF166).

**Figure 6:**
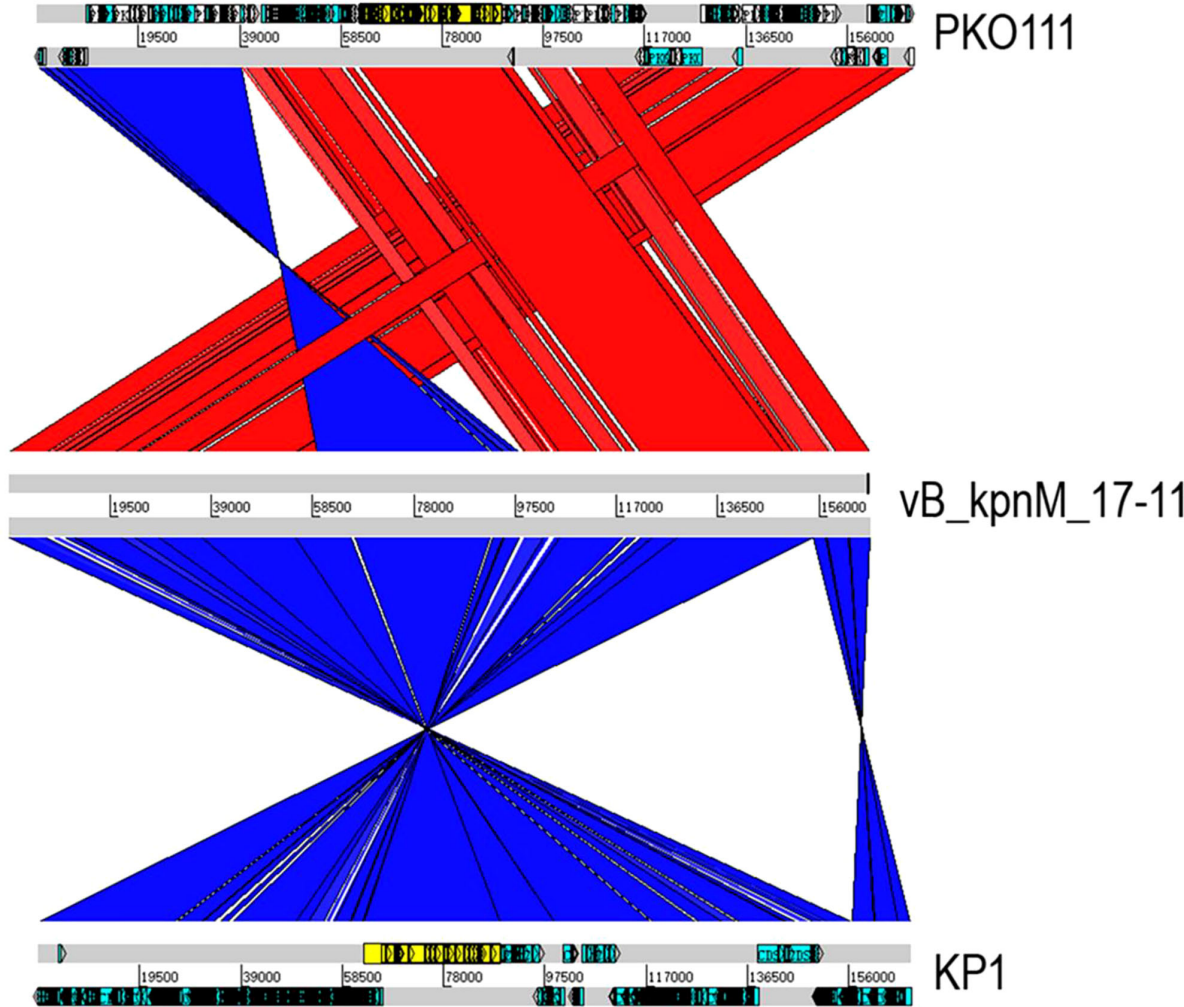
Multiple genome alignments of phage vB_kpnM_17-11, PKO111 and KP1. Red and blue represent the forward and reverse matches, respectively. The darker the color, the higher similarity.

**Figure 7:**
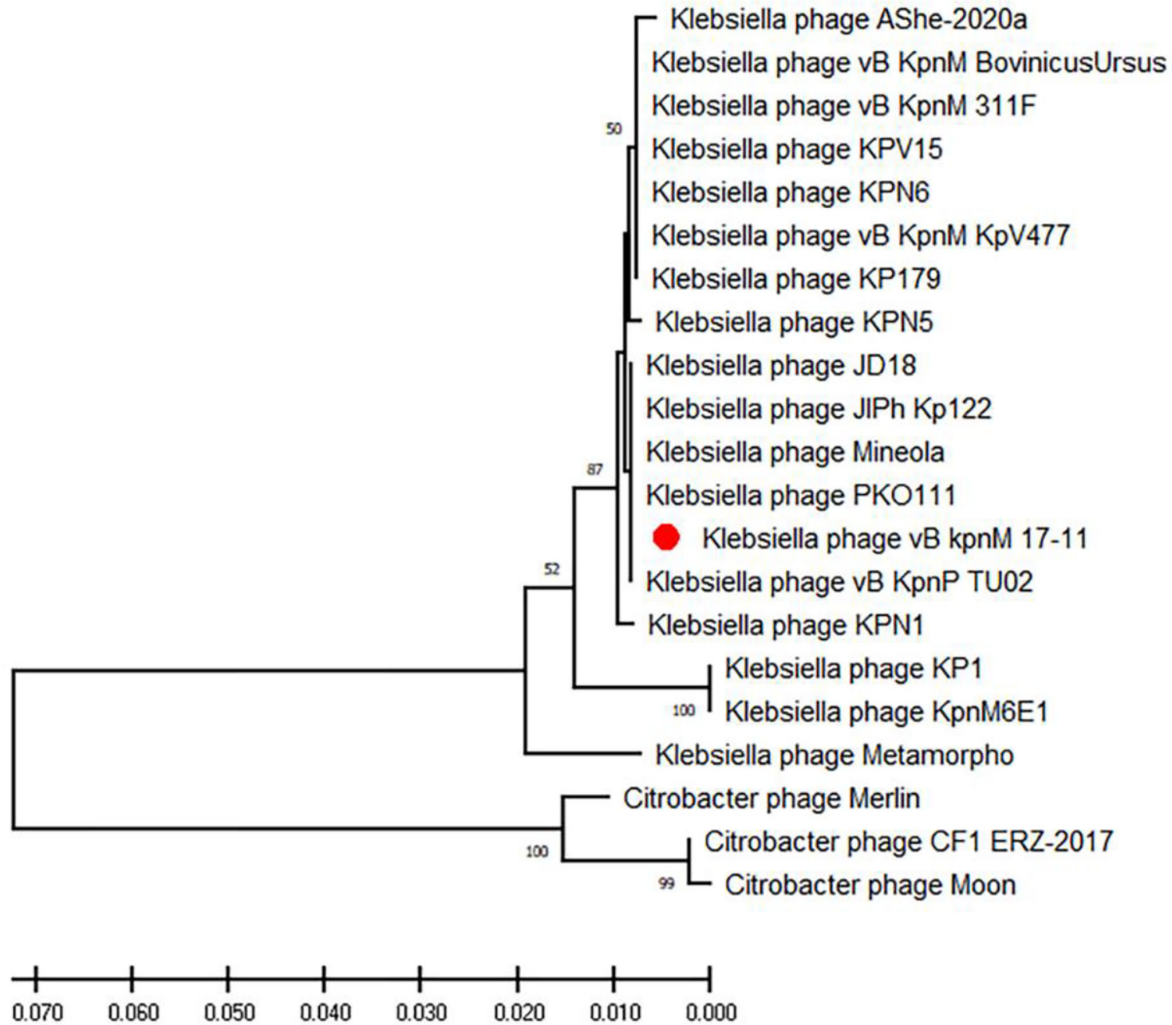
Phylogenetic tree of vB_kpnM_17-11 based on MCP. Phylogenetic tree of phages established using MEGA-X software by the neighbor-joining method with 1000 bootstrap replicates. The red dot highlights the vB_kpnM_17-11.

The genome of vB_kpnM_17-11 shows high identity (> 90%) with the genomes of other 18 *Klebsiella* phages (**Table 2**). *Klebsiella* phages PKO111 and KP1 were used for multiple sequence alignment with vB_kpnM_17-11. Thus, vB_kpnM_17-11 has similar genome sequence and gene module arrangement as *Klebsiella* phages PKO111 and KP1. However, each phage has a distinct genomic organization (**Figure 6**). To determine the evolutionary relationships of phage vB_kpnM_17-11 with respect to other *Klebsiella* phages, a phylogenetic tree based on MCP was constructed. The phylogenetic reconstruction demonstrated that phage vB_kpnM_17-11 has a close evolutionary relationship with a variety of *Klebsiella* phage, such as PKO111, vB_kpnP_TU02, JD18, JIPh Kp122 and Mineola (**Figure 7**). Phage morphology and whole genome indicate that vB_kpnM_17-11 belongs to the family of *Myoviridae*, order *Caudovirales*.

**Table 2:** Global genome comparison of vB_kpnM_17-11 with homologous phages.

	Host strain type	GenBank number	Genome size (bp)	Query coverage with vB_kpnM_17-11	Identity with vB_kpnM_17-11
vB_kpnM_17-11	*K. pneumoniae*	MW239157	165894 bp	100%	100%
PKO111	*K. oxytoca*	KR269720	168758 bp	94%	96.60%
KP1	*K. pneumoniae*	MG751100	167989 bp	94%	96.70%
KPV15	*K. pneumoniae*	NC_055715	167034 bp	93%	96.79%
vB_KpnM_311F	*Klebsiella*	LR877331	166710 bp	96%	95.17%
KPN6	*K. pneumoniae* *K. oxytoca*	MN101230		94%	95.08%
KPN5	*K. pneumoniae* *K. oxytoca*	MN101229	158756 bp	91%	95.04%
vB_KpnM_BovinicusUrsus	*K. pneumoniae*	MW021752	166829 bp	95%	94.52%
Mineola	*K. pneumoniae*	NC_055748	166130 bp	93%	94.24%
KP179	*K. pneumoniae*	NC_055768	162630 bp	91%	96.55%
KPN2 HKu-2019	*K. pneumoniae* *K. oxytoca*	MN101226	122724 bp	67%	95.00%
JD18	*K. pneumoniae*	KT239446	166313 bp	95%	96.03%
JIPh_Kp122	*K. pneumoniae*	MN434095	166475 bp	95%	95.99%
KpnM6E1	*K. pneumoniae*	MT580897	167920 bp	94%	96.56%
AShe-2020a	*K. pneumoniae*	CP062992	168979 bp	93%	96.10%
KPN1	*K. pneumoniae* *K. oxytoca*	MN101225	98975 bp	56%	95.71%
vB_KpnM_KpV477	*K. pneumoniae*	KX258185	168272 bp	93%	94.13%
vB_KpnP_TU02	*K. pneumoniae*	MZ560702	166230 bp	93%	97.53%
Metamorpho	*K. aerogenes*	MT701588	171475 bp	77%	91.18%

### Analysis of Depolymerase Dep022 Coding Gene

**Figure 8:**
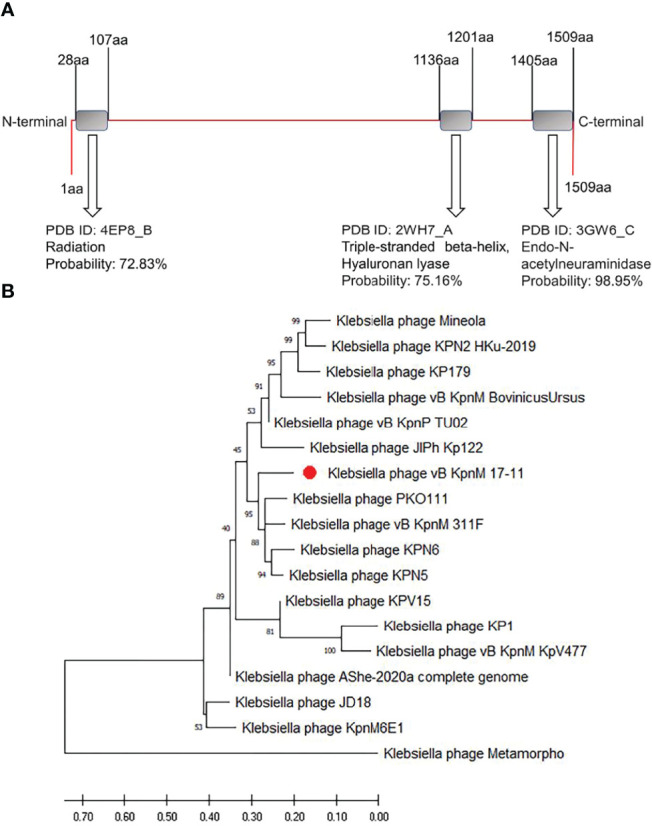
The prediction domines and phylogenetic tree of depolymerase Dep022. **(A)** Domains of depolymerase Dep022 predicted using HHpred software. HHpred prediction results show that DEP022 has three different domains. **(B)** Phylogenetic tree of depolymerase. Phylogenic tree of depolymerases established using MEGA-X software by the neighbor-joining method with 1000 bootstrap replicates. The red dot highlights the Dep022 depolymerase of vB_kpnM_17-11.

BLAST results showed that 99.08% (108/109) of vB_kpnM_17-11 functional genes had more than 95% similarity to known homologues in other phages. However, *orf022* had only 85.97% similarity with the L-shaped tail fiber protein of *Klebsiella* Phage vB_KpnM_311F. This suggests that this protein of phage vB_kpnM_17-11 is rather distinct. The size of *orf022* is 4530 bp and encodes a 1509 aa protein, annotated as a L-shaped tail fiber protein. HHpred software predicted this protein to contain two domains of depolymerase activity, namely a domain with a hyaluronidase function (PDB ID: 2WH7_A, probability = 72.83%) and a domain with Endo-N-acetylneuraminidase function (PDB ID: 3GW6_C, probability = 98.95%) analogous to those of *Escherichia coli* phage K1F (**Figure 8A**). Thus, *orf022* may encode a depolymerase protein, which we named Dep022. Dep022 has the closest evolutionary relationship with the proteins encoded by *Klebsiella* phage KPO111, vB_KpnM_311F, KPN6 and KPN5 (**Figure 8B**).

### Antibacterial Activity *in Vitro* and *in Vivo*

**Figure 9:**
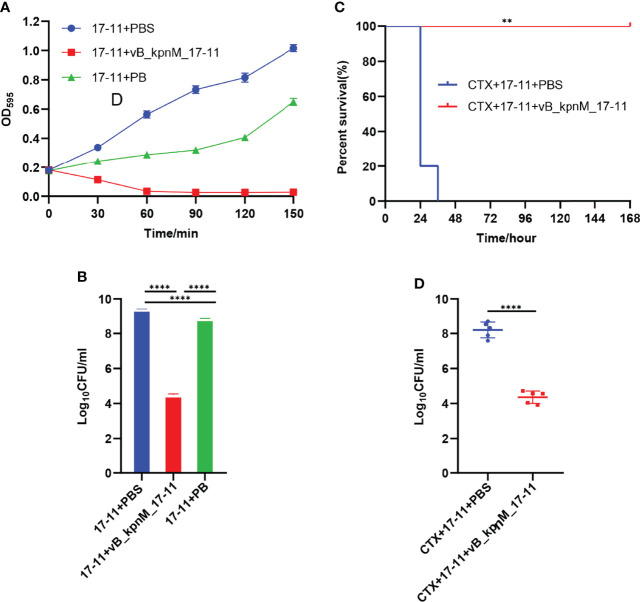
Sterilization experiment *in vitro* and *in vivo*. **(A)** Bacteriostatic curve of phage *in vitro*. PBS, vB_kpnM_17-11 or PB were added to 50 mL exponential phase *K. pneumoniae* 17-11. The value of OD_595_ was measured every 30 min from 0 min to 180 min. **(B)** Bactericidal efficiency of phage *in vitro*. ****P < 0.0001, two-sided Mann–Whitney test. **(C)** Survival of mice after infection by *K. pneumoniae* 17-11. **P < 0.01 (Gehan-Breslow-Wilcoxon test). **(D)** Abdominal lavage fluid bacterial load. Mice were infected with *K. pneumoniae* 17-11 intraperitoneally and treated with phage. Peritoneal lavage was harvested at 24 h post infection and CFU were quantified. ****P < 0.0001, two-sided Mann–Whitney test.

vB_kpnM_17-11 was able to effectively inhibit the growth of 17-11 (**Figure 9A**). Bacterial growth in the 17-11+PBS control group was not inhibited as determined by OD_595_ recording. The OD_595_ values in the 17-11+vB_kpnM_17-11 group decreased with time, dropping to baseline levels after 60 min. Bacterial growth in the presence of PB was notably inhibited for 120 min, but a trend towards a steep increase in bacterial numbers was recorded at the last time point (150 min), suggesting that bacteria were able to overcome the action of the antibiotic. The content of bacteria in 17-11+PBS group, 17-11+vB_kpnM_17-11 group and 17-11+PB group was 2.44 × 10^9^ CFU/mL, 3.10 × 10^4^ CFU/mL and 7.07 × 10^8^ CFU/mL, respectively. The bacterial count of the 17-11+vB_kpnM_17-11 group was significantly lower than that of 17-11+PBS group and 17-11+PB group. At the later stage of the experiment, bacterial counts in the 17-11+PB group began to increase, but the count was significantly lower than of the 17-11+PBS group (**Figure 9B**). Compared with the 17-11+PBS group, vB_kpnM_17-11 *in vitro* reduced bacteria by 10^5^ times from 10^9^ to 10^4^ CFU/mL.

To test the performance of vB_kpnM_17-11 *in vivo*, experiments were performed in a mouse infection model. The survival rate of mice in the CTX+17-11+PBS group was 20% within 24 h, and all mice died within 36 h. However, all mice in the CTX+17-11+vB_kpnM_17-11 group survived (**Figure 9C**). Bacterial cell counts in mice peritoneal lavage fluids were 2.27 × 10^8^ CFU/mL for the CTX+17-11+PBS group, and 2.89 × 10^4^ CFU/mL for the CTX+17-11+vB_kpnM_17-11 group. Therefore, vB_kpnM_17-11 reduced the bacterial content of peritoneal lavage fluid by 10^4^ times in immunodeficient mice (**Figure 9D**).

## Discussion

An increasing number of CRKP isolates poses a significant challenge to public health worldwide (Cai et al., 2019; Zhang et al., 2022). The safety and effectiveness of phages and their coding products in the treatment of drug-resistant bacterial infections have been extensively demonstrated (Majkowska-Skrobek et al., 2018). Therefore, research and development of novel therapies based on phages and their coding products may help treat infections caused by drug-resistant bacteria, which are on the rise and expected to increase in the coming years. In this study, we isolated a phage vB_kpnM_17-11 that can lyse CRKP, studied its biological characteristics, analyzed its whole genome and coding products, and studied its efficacy *in vitro* and *in vivo*.

vB_kpnM_17-11 can form transparent plaques with a halo of around of 3 mm in diameter, and the plaque indicates that vB_kpnM_17-11 is a lytic phage (Peng et al., 2020). As a lytic phage, vB_kpnM_17-11 has promising potential prospects in clinical therapy and production of phage preparations. When bacterial growth on the plate reaches a plateau, phage proliferation usually stops or slows down dramatically, resulting in no increase in the plaque area. However, the tails of some phages can still depolymerize bacterial exopolysaccharides, resulting in translucence regions of varying sizes on the outside of the phage plaque, called halos (Cornelissen et al., 2011). The presence of a halo indicates that the phage has depolymerase activity, and depolymerase is a potential choice for treating antibiotic-resistant bacterial infections (Dunstan et al., 2021). Compared to literature data, the plaque size of vB_kpnM_17-11 and TSK1 were similar, but the halo of *Klebsiella* phage TSK1 and *Klebsiella* phage π VLC6 were larger than that vB_kpnM_17-11, suggesting that TSK1 (Tabassum et al., 2018) and π VLC6 (Domingo-Calap et al., 2020) might have more powerful depolymerase activity. Genome sequence analysis in combination with the study of morphological features such as the retractable tail structure of vB_kpnM_17-11 indicate that the phage belongs to the *Myoviridae* family, order *Caudovirales*.

The optimal MOI for phages varies, and the smaller the optimal MOI, the fewer phages required to lyse the same number of bacteria. The optimal MOI of vB_kpnM_17-11 was 0.001, which was similar to that reported for *Klebsiella* phage vB_KpnP_IME337 (Gao et al., 2020), indicating that when their MOI was 0.001, the unit phage produced the most progeny and had the highest proliferation efficiency. In the industrial preparation of phages, to obtain a higher titer, researchers often culture phages based on the optimal MOI, which reduces production costs and increases profits. The growth curve of phage is divided into latency phase, logarithmic phase and stationary phase. The incubation period of vB_kpnM_17-11 was 30 min, in contrast with that of the *Klebsiella* phage vB_KleS-HSE3 which was 40 min (Peng et al., 2020). Phages with shorter incubation periods can produce progeny faster and increase the number of phages in a shorter time. Furthermore, the long logarithmic period of the vB_kpnM_17-11 may be related to its larger whole genome and longer gene replication time.

The titer of vB_kpnM_17-11 remains stable at temperature between 4°C and 40°C and pH between 4 - 10. This temperature and pH range are just the common environment for phage preparation production, transportation, storage and treatment, indicating that the phage has the potential for further industrial application (Jończyk et al., 2011). Furthermore, phage with versatile temperature and pH adaptability are more suitable for various infection models, such as skin infections that become more alkaline due to bacterial colonization, or urinary tract infections that have highly variable microenvironments (Martín-Rodríguez et al., 2020). The loss of viability of t vB_kpnM_17-11 at pH = 1 prevents its oral administration because of the acidic conditions of the gastric environment (Beasley et al., 2015). To overcome this drawback, vB_kpnM_17-11 can be protected by alginate and chitosan to avoid inactivation by gastric juices (Silva Batalha et al., 2021). A limitation of vB_kpnM_17-11 is its narrow range, being able to lyse only 4 out of 96 tested strains of *K. pneumoniae*. Lysed strains belonged to serotype K19. In this sense, phages with a wider host range are desirable. In addition, bacteria are more likely to develop phage resistance to narrow host spectrum phages. These limitations should be acknowledged when considering future applications of vB_kpnM_17-11. On the other hand, however, phages that lyse single bacterial serotypes may prove useful in bacterial serotyping (Song et al., 2021). Differences in host range are often related to the phage tail structure responsible for recognition and adsorption. However, the specific mechanism of host range of vB_kpnM_17-11 needs to be further studied.

The basic characteristics of the vB_kpnM_17-11 genome are similar to those of other published *Myoviridae* phages. Their genomes are all dsDNA and the genes encoding the same function are arranged in clusters (Dion et al., 2020). The proportion of non-coding regions of vB_kpnM_17-11 is only 3%. The coding genes for vB_kpnM_17-11 are tightly packed, with overlapping ORFs. This phenomenon allows phages to encode enough functional genes in a small genome; the smaller the genome, the lower the cost of replication.

vB_kpnM_17-11 genome was modularized in this study. The nucleotide metabolism and replication module is mainly responsible for the replication and regulation of phage genome. Usually, dsDNA phage genomes encode their own RNA polymerase (RNAP) (Zhao et al., 2019), but the phage vB_kpnM_17-11 does not encode RNAP. In addition, we found sigma factors and RNA polymerase binding proteins in the vB_kpnM*_*17-11 genome, which can modify host RNAP to recognize the promoter region of phage genes to complete the expression of phage genes (Yuan et al., 2020). In the packaging module, a putative portal vertex protein is predicted to form phage DNA channels into empty capsid proteins located at the tip of the phage head. The terminal enzyme large subunit transfers the phage DNA to the empty capsid and modifies it to complete the packaging process. The small subunit of the terminal enzyme binds to the packaging initiation site and regulates the ATP activity of the large subunit of the terminal enzyme (Feiss and Rao, 2012). In the host lysis module, the RZ/RZ1 protein widely present in Gram-negative phages was also found in vB_kpnM_17-11. This protein has certain peptidase activity, which can degrade the oligopeptide bond between the cell wall and the cell membrane, and promote the rapid release of daughter phages. dsDNA phages are mostly Holin-Endolysin systems, but vB_kpnM_17-11 has only holin (Young et al., 2000); endolysin is not found. This suggests that the phage may encode a new endolysin or have other lysing systems, which need to be further studied. The genes in the morphological module can be divided into two parts, the head morphological genes and the tail morphological genes. The genes responsible for phage head formation tend to be highly conserved to ensure the life process of the phage, but the phage tail genes experience frequent mutations to change their structure to recognize more diverse bacteria (Dion et al., 2020).

Multiple sequence alignment and BLAST highlighted the similarity between vB_kpnM_17-11 and related phages such as PKO111 and KP1. The genes with lower similarity were mainly phage tail genes. The tail gene of the phage is most prone to mutation and evolution (Comeau et al., 2007). The tail genes are often related to recognition and adsorption, so the phage gene rearrangement and mutation may affect the host range of the phage. This may be the reason why vB_kpnM_17-11 has a high similarity with PKO111 and KP1 genes, but the host range is completely different. Phylogenetic analysis showed that vB_kpnM_17-11 was closely related to *Klebsiella* phages PKO111, vB_kpnP_TU02, JD18, JIPh Kp122 and Mineola. Genomic comparisons also showed that these phages were similar and evolutionarily close to vB_kpnM_17-11.

Depolymerase was first reported in the 1956s (Adams and Park, 1956). Specifically, the depolymerase encoded by *Pseudomonas aeruginosa* phage IME180 (Mi et al., 2019), *Escherichia coli* phage vB_EcoM_ECOO78 (Guo et al., 2017) and *K. pneumoniae* phage KP36 (Majkowska-Skrobek et al., 2016) showed great biofilm elimination and antibacterial ability *in vitro*. In our study, we found a phage depolymerase Dep022. Dep022 contains two depolymerase domains and shares an ORF with the phage structural protein. The Endo-N-acetylneuraminidase domain of Dep022 belongs to the hydrolytic depolymerase domain, which acts by hydrolyzing the side chain of the O-antigen of lipopolysaccharide (LPS) or the oxyglycoside bond in the capsular polysaccharide (Kim et al., 2011). Neuraminidase is the most common structural protein depolymerase in phages and is widely distributed in the tail fiber and tail plate of *Myoviridae* phages (Pires et al., 2016). The hyaluronidase domain belongs to the lytic depolymerase domain, which cleaves polysaccharides into monosaccharides by directly severing the chemical bond between monosaccharides and C4 uronic acids and introducing unsaturated bonds between the aldehyde acid ends of C4 and C5. However, the related domain of hyaluronidase is relatively rare in phages (Pires et al., 2016). At the same time, the two depolymerase domains presented us with a problem: we were unable to determine the type of Dep022. The resolution of this problem depends on the subsequent study of Dep022. In addition, DepoKP36 (Majkowska-Skrobek et al., 2016), a depolymerase encoded by *K. pneumoniae* phage KP36 with a similar Endo-N-acetylneuraminidase domain, has been confirmed to have depolymerase activity. This finding further raises the possibility that Dep022 has depolymerase activity. Phylogenetic tree analysis showed that Dep022 had a close genetic relationship with other similar phage genes that might encode depolymerases. Nonetheless, these phages have not been reported to have depolymerase activity, which may be related to gene point mutations in the phage genes.

PB and vB_kpnM_17-11 were used in *in vitro* sterilization tests. We found that vB_kpnM_17-11 is more effective than PB in terms of bactericidal ability, which may be associated with the continuous reproduction of vB_kpnM_17-11 (Taati Moghadam et al., 2020). vB_kpnM_17-11 reduced the number of bacteria *in vitro* from 10^9^ CFU/mL to 10^4^ CFU/mL, a 10^5^-fold reduction. In comparison, phage phiLLS could only lyse 94% of *Escherichia coli in vitro* at MOI of 10 and 100 (Amarillas et al., 2017), indicating that the lytic efficiency of vB_kpnM_17-11 was higher than that of phiLLS. vB_kpnM_17-11 was able to increase the 24 hours survival rate of systemic-infected mice from 20% to 100%, and the therapeutic effect was comparatively better than that reported for *Klebsiella* phage 1513 (Cao et al., 2015) which can improve the 24 hours survival rate of mice with pneumonia by 70%. vB_kpnM_17-11 reduced the number of bacteria in peritoneal lavage fluid by 10^4^ times, while *Klebsiella* phage 1513 only reduced bacterial load in the lungs of mice by 10^2^ times (Cao et al., 2015). However, the phage cocktail VBSM-A1/VBSP-A2 (Geng et al., 2020) reduced bacterial load in the breast of mice about 10^5^ times, which was superior to vB_kpnM_17-11. Frequently, phage cocktails work better than therapies based on a single phage. Furthermore, phage treatment has also shown great therapeutic effects in human. Recently, researchers cleared CRKP from the patients’ intestines using oral and rectal administration of phage preparations (Corbellino et al., 2020). In addition to clinical treatment, phage preparations are also expected to play a key role in environmental disinfection, fisheries, animal husbandry, and industrial fermentation. Phages show good antibacterial effects as potential antibacterial agents, and their effects on bacteria deserve further study.

Previous studies have also isolated phages that lyse CRKP, such as kpssk3 (Shi et al., 2020), vB_KpnP_IME337 (Gao et al., 2020), and TSK1 (Tabassum et al., 2018). The biological characteristics of vB_kpnM_17-11 were not significantly different from those of these phages in terms of the one-step growth curve, temperature, and pH stability. In terms of genetic characteristics, their genome size and ORF number vary greatly, which is related to the large difference in phage genome among different species. The biggest difference was in the host range, kPSSK3 could lyse 25 of 77 strains, vB_KpnP_IME337 had no sensitivity to other 29 strains except host bacteria, and vB_kpnM_17-11 could lyse the only 4 of 92 strains. The difference in host range is related to the difference in phage tail structure, and the specific mechanism remains to be further explored. But vB_kpnM_17-11 has two potential advantages over other phages that can lyse CRKP. One advantage is that we can develop new antibacterial agents based on the predicted depolymerase Dep022, which is expected to ameliorate the increasingly serious bacterial resistance phenomenon. Another advantage is that we can use more *K. pneumoniae* to explore the host range of vB_kpnM_17-11. If vB_kpnM_17-11 is specific for *K. pneumoniae* serotype K19, then vB_kpnM_17-11 has potential application in the identification of *K. pneumoniae* serotype.

Although phages have broad clinical application prospects for the treatment of infections caused by drug-resistant bacteria, phages still have limitations in practical clinical application. First, the *in vivo* pharmacokinetics and pharmacodynamics of different phage preparations vary compared to antibiotic therapy. Preparations containing different phages have different biological properties, so there are many differences in practical clinical applications (Nilsson, 2019). In addition, there are no standard treatment guidelines for phage therapy, and there are no effective guidelines for how much phage should be used during treatment, how long it should be administered, and how it should be administered. Furthermore, certain endotoxins are produced during the production of phage preparations, which may be cytotoxic and immunogenic. According to the upper limit of endotoxin content of existing clinical preparations of 5 EU/(kg·h), most phage preparations do not meet the standards for clinical use (Henein, 2013). Finally, phage therapy may not be accepted by some people due to the lack of awareness of phages among medical personnel and the public. Although there are still many problems in the large-scale clinical application of phages, more and more researchers have paid attention to the value of phage therapy. It is important to note that the use of phages is not only the use of phages to treat bacterial infections, but also includes the use of phage antibiotic combinations, phage-derived enzymes and phage disinfection in hospital environments (Gordillo Altamirano and Barr, 2019). It is believed that with the further development of phage research, the clinical application of phage will usher in a bright future (Sharma et al., 2017).

## Data Availability Statement

The datasets presented in this study can be found in online repositories. The names of the repository/repositories and accession number(s) can be found below: https://www.ncbi.nlm.nih.gov/nuccore/MW239157.

## Ethics Statement

The animal study was reviewed and approved by Ethics Committee of Experimental Animals, Southwest Medical University 20211124-002.

## Author Contributions

JB, RH and YZ conceived and designed this study. JB performed the experiments and drafted the manuscript. FZ, SL, QC, WW and RH contributed reagents/materials/analysis tools. YW, AM-R, ÅS and YZ critically revised the manuscript. All authors read and approved the final manuscript. All authors contributed to the article and approved the submitted version.

## Funding

This research was funded by the National Natural Science Foundation of China [31500114], the Sichuan Province Science and Technology project [2020YJ0338], Natural Science Foundation of Luzhou (2021-NYF-20) and Southwest Medical University project [21YYJC0529].

## Conflict of Interest

The authors declare that the research was conducted in the absence of any commercial or financial relationships that could be construed as a potential conflict of interest.

## Publisher’s Note

All claims expressed in this article are solely those of the authors and do not necessarily represent those of their affiliated organizations, or those of the publisher, the editors and the reviewers. Any product that may be evaluated in this article, or claim that may be made by its manufacturer, is not guaranteed or endorsed by the publisher.
