# Supplementary material for: Isolation and Characterization of vB_kpnM_17-11, a Novel Phage Efficient Against Carbapenem-Resistant Klebsiella pneumoniae
Source: Front Cell Infect Microbiol. 2022 Jul 5;12:897531. doi: 10.3389/fcimb.2022.897531 (PMC9294173; doi:10.3389/fcimb.2022.897531)
Supplement: Supplementary file 1 [file DataSheet_1.docx]

**Supplementary Table 1.** MIC of *K. pneumoniae* 17-11.

| Antimicrobial drugs name | MIC (ug/mL) | Result |
| --- | --- | --- |
| Cefotaxime | 128 | R |
| Ceftazidime | 64 | R |
| Ceftizoxime | 64 | R |
| Meropenem | 16 | R |
| Imipenem | 64 | R |
| Polymyxin B | 2 | S |
| Chloramphenicol | 16 | I |
| Tetracycline | 512 | R |

Note: S: Susceptible; I: Intermediary resistant; R: Resistant.


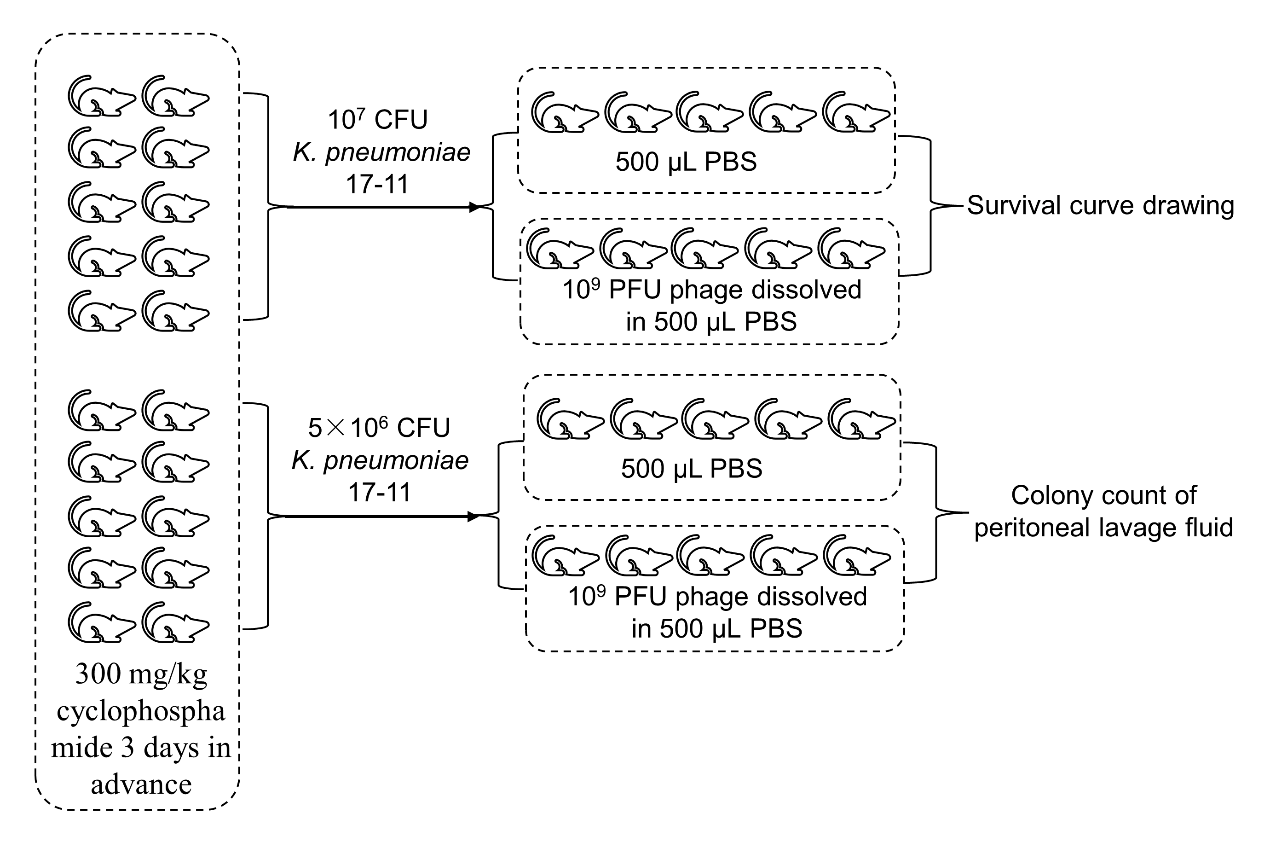


**Supplementary Figure. 1** **Flow chart of *in vivo* bacteriostatic experiment.** The phage *in vivo* experiment was divided into two parts, respectively to explore the survival curve of mice and the number of bacteria in the mice's abdominal cavity.

**Supplementary Table. 2 Host range of phage vB_kpnM_17-11.** 96 strains of bacteria were used to test the host range of vB_kpnM_17-11.

| Bacterium | Plaque | Bacterium | Plaque |
| --- | --- | --- | --- |
| *K. pneumoniae* 17-11 | + | *K. pneumoniae* B8 | + |
| *K. pneumoniae* H27 | + | *K. pneumoniae* S43 | + |
| *Pseudomonas aeruginosa* ATCC 27853 | － | *Pseudomonas aeruginosa* PA05 | － |
| *Pseudomonas aeruginosa* PA14 | － | *Pseudomonas aeruginosa PA29* | － |
| *Pseudomonas aeruginosa PA46* | － | *Escherichia coli* ATCC 25922 | － |
| *Escherichia coli* E5 | － | *Escherichia coli* E8 | － |
| *Escherichia coli* E17 | － | *Escherichia coli* E37 | － |
| *Salmonella* CMCC 50761 | － | *Salmonella* SJ42 | － |
| *Salmonella* SJ56 | － | *Salmonella* SJ60 | － |
| *Salmonella* SJ11 | － | *Staphylococcus aureus* ATCC 29213 | － |
| *K. pneumoniae* ATCC700603 | － | *K. pneumoniae* S6 | － |
| *K. pneumoniae* S5 | － | *K. pneumoniae* S7 | － |
| *K. pneumoniae* S4 | － | *K. pneumoniae* S9 | － |
| *K. pneumoniae* 5 | － | *K. pneumoniae* S10 | － |
| *K. pneumoniae* 7 | － | *K. pneumoniae* S12 | － |
| *K. pneumoniae* 28 | － | *K. pneumoniae* S13 | － |
| *K. pneumoniae* 42 | － | *K. pneumoniae* S14 | － |
| *K. pneumoniae* 48 | － | *K. pneumoniae* S16 | － |
| *K. pneumoniae* B1 | － | *K. pneumoniae* S17 | － |
| *K. pneumoniae* B2 | － | *K. pneumoniae* S19 | － |
| *K. pneumoniae* B3 | － | *K. pneumoniae* S20 | － |
| *K. pneumoniae* F6 | － | *K. pneumoniae* S21 | － |
| *K. pneumoniae* F7 | － | *K. pneumoniae* S24 | － |
| *K. pneumoniae* F9 | － | *K. pneumoniae* S25 | － |
| *K. pneumoniae* F11 | － | *K. pneumoniae* S26 | － |
| *K. pneumoniae* F12 | － | *K. pneumoniae* S27 | － |
| *K. pneumoniae* F16 | － | *K. pneumoniae* S28 | － |
| *K. pneumoniae* F23 | － | *K. pneumoniae* S29 | － |
| *K. pneumoniae* F25 | － | *K. pneumoniae* S32 | － |
| *K. pneumoniae* F26 | － | *K. pneumoniae* S33 | － |
| *K. pneumoniae* F32 | － | *K. pneumoniae* S35 | － |
| *K. pneumoniae* F38 | － | *K. pneumoniae* S38 | － |
| *K. pneumoniae* F42 | － | *K. pneumoniae* S39 | － |
| *K. pneumoniae* F43 | － | *K. pneumoniae* 0867 | － |
| *K. pneumoniae* F44 | － | *K. pneumoniae* 1105 | － |
| *K. pneumoniae* F45 | － | *K. pneumoniae* 1198 | － |
| *K. pneumoniae* F46 | － | *K. pneumoniae* 1373 | － |
| *K. pneumoniae* F48 | － | *K. pneumoniae* 2260 | － |
| *K. pneumoniae* F91 | － | *K. pneumoniae* 2394 | － |
| *K. pneumoniae* F97 | － | *K. pneumoniae* 2421 | － |
| *K. pneumoniae* F100 | － | *K. pneumoniae* 2466 | － |
| *K. pneumoniae* F102 | － | *K. pneumoniae* 2583 | － |
| *K. pneumoniae* F104 | － | *K. pneumoniae* 2673 | － |
| *K. pneumoniae* F105 | － | *K. pneumoniae* 2751 | － |
| *K. pneumoniae* F106 | － | *K. pneumoniae* 3041 | － |
| *K. pneumoniae* F107 | － | *K. pneumoniae* 3063 | － |

+: vB_kpnM_17-11 can form plaque; －: vB_kpnM_17-11 cannot form plaque

**Supplementary Table. 3** **List of the predicted ORFs in the genome of phage vB_kpnM_17-11.**

| **ORFs** | **Start** | **End** | **Annotation** | **Identity (%)** | **Accession number** |
| --- | --- | --- | --- | --- | --- |
| 001 | 165457 | 165894 | Endonuclease | 536/549(98%) | KR269720.1 |
|  | 1 | 111 |  |  |  |
| 002 | 175 | 414 | Hypothetical protein | 239/240(99%) | KX258185.1 |
| 003 | 476 | 592 | Hypothetical protein | 116/117(99%) | MN101226.1 |
| 004 | 603 | 794 | Hypothetical protein | 187/192(97%) | MG751100.1 |
| 005 | 791 | 880 | Hypothetical protein | 90/90(100%) | KR269720.1 |
| 006 | 939 | 1394 | Hypothetical protein | 451/456(99%) | MG751100.1 |
| 007 | 1412 | 1567 | Hypothetical protein | 156/156(100%) | MN434095.1 |
| 008 | 1564 | 1704 | Hypothetical protein | 140/141(99%) | MH333064.1 |
| 009 | 1710 | 3098 | DNA topoisomerase | 1327/1389(96%) | MG751100.1 |
| 010 | 3325 | 3588 | Hypothetical protein | 261/264(99%) | MN434095.1 |
| 011 | 3712 | 4356 | Putative transcriptional regulator MotA | 632/645(98%) | KR269720.1 |
| 012 | 4356 | 4697 | Hypothetical protein | 340/342(99%) | KT239446.1 |
| 013 | 4694 | 5107 | Hypothetical protein | 391/426 (92%) | KR269720.1 |
| 014 | 5107 | 5409 | Hypothetical protein | 303/303(100%) | KR269720.1 |
| 015 | 5390 | 5515 | Hypothetical protein | 113/125(90%) | MK421971.1 |
| 016 | 5499 | 5777 | Hypothetical protein | 270/279(97%) | MH333064.1 |
| 017 | 5774 | 6100 | Hypothetical protein | 319/326(98%) | KR269720.1 |
| 018 | 6097 | 6363 | Hypothetical protein | 263/267(99%) | KR269720.1 |
| 019 | 6431 | 6703 | Anti-sigma factor | 272/273(99%) | MK421971.1 |
| 020 | 6737 | 7360 | Putative holin | 617/624(99%) | MN101229.1 |
| 021 | 7390 | 7929 | Distal long tail fiber assembly catalyst | 518/540(96%) | MN101229.1 |
| 022 | 7945 | 12474 | L-shaped tail fiber protein | 2478/2880(86%) | MN101229.1 |
| 023 | 12484 | 13146 | Tail fibers protein | 646/663(97%) | MN101230.1 |
| 024 | 13204 | 17049 | Long tail fiber proximal subunit | 3746/3846(97%) | MN101229.1 |
| 025 | 17049 | 18185 | Phage tail fiber protein (long tail fiber) | 1109/1137(98%) | KR269720.1 |
| 026 | 18261 | 19187 | Phage ribonuclease H | 892/927(96%) | MG751100.1 |
| 027 | 19198 | 19470 | DsbA dsDNA binding protein | 270/273 (99%) | MG751100.1 |
| 028 | 19451 | 19750 | Transcriptional regulator | 294/300(98%) | KR269720.1 |
| 029 | 19747 | 20409 | Helicase loading protein | 661/663(99%) | MN101230.1 |
| 030 | 20477 | 21394 | Putative single-stranded DNA binding protein | 911/918(99%) | MN101226.1 |
| 031 | 21522 | 21779 | Hypothetical protein | 258/258 (100%) | KR269720.1 |
| 032 | 21839 | 22117 | Hypothetical protein | 278/279 (99%) | MG751100.1 |
| 033 | 22114 | 22203 | Hypothetical protein | 90/90 (100%) | MK421971.1 |
| 034 | 22196 | 22516 | Hypothetical protein | 312/321 (97%) | KR269720.1 |
| 035 | 22599 | 22763 | Hypothetical protein | 160/165 (97%) | MN434095.1 |
| 036 | 22771 | 23370 | Putative dihydrofolate reductase | 583/600 (97%) | MH729874.1 |
| 037 | 23354 | 23671 | Hypothetical protein | 312/318 (98%) | KR269720.1 |
| 038 | 23664 | 24524 | Putative thymidylate synthase | 821/857 (96%) | KR269720.1 |
| 039 | 24521 | 24727 | Hypothetical protein | 180/207 (87%) | MN101226.1 |
| 040 | 24724 | 25029 | Phage protein | 301/306 (98%) | MK421971.1 |
| 041 | 25020 | 27266 | Ribonucleotide reductase | 2163/2247 (96%) | MH729874.1 |
| 042 | 27323 | 28465 | Ribonucleotide reductase | 1126/1143(99%) | KR269720.1 |
| 043 | 28493 | 28909 | Endonuclease | 411/417 (99%) | MH333064.1 |
| 044 | 28903 | 30054 | RNA ligase | 1122/1152(97%) | MG751100.1 |
| 045 | 30117 | 30626 | RNA ligase | 493/510 (97%) | MN101225.1 |
| 046 | 30614 | 30967 | Hypothetical protein | 341/354(96%) | MN434095.1 |
| 047 | 30964 | 31251 | Phage outer membrane lipoprotein Rz1 | 288/288(100%) | MH729874.1 |
| 048 | 31274 | 31759 | Hypothetical protein | 479/486(99%) | MH729874.1 |
| 049 | 31759 | 31971 | Hypothetical protein | 213/213(100%) | MH729874.1 |
| 050 | 31971 | 32864 | 3'-phosphatase, 5'-polynucleotide kinase,phage associated | 888/894(99%) | MH729874.1 |
| 051 | 32876 | 33160 | Hypothetical protein | 284/285(99%) | MH729874.1 |
| 052 | 33150 | 33386 | Hypothetical protein | 235/237(99%) | MN101230.1 |
| 053 | 33386 | 33730 | Hypothetical protein | 338/345(98%) | KR269720.1 |
| 054 | 33731 | 34300 | dCMP deaminase | 568/570(99%) | KR269720.1 |
| 055 | 34300 | 34500 | Hypothetical protein | 201/201(100%) | MH729874.1 |
| 056 | 34497 | 34805 | Phage tail fibers | 306/309(99%) | MN101230.1 |
| 057 | 34863 | 35186 | Hage head assembly chaperone protein | 320/324(99%) | MN101225.1 |
| 058 | 35302 | 35550 | Lysis inhibition accessory protein | 249/249(100%) | LR877331.1 |
| 059 | 35919 | 36095 | Hypothetical protein | 177/177(100%) | MN434095.1 |
| 060 | 36217 | 36585 | Hypothetical protein | 353/370(95%) | MG751100.1 |
| 061 | 36624 | 37133 | Hypothetical protein | 398/516(77%) | LR877331.1 |
| 062 | 37227 | 37586 | Hypothetical protein | 358/360(99%) | KY000080.1 |
| 063 | 37626 | 38108 | Hypothetical protein | 480/483(99%) | MN101230.1 |
| 064 | 38121 | 38399 | Hypothetical protein | 271/279(97%) | KR269720.1 |
| 065 | 38410 | 38580 | Hypothetical protein | 169/171(99%) | MH333064.1 |
| 066 | 38577 | 38777 | Hypothetical protein | 197/201(98%) | MH333064.1 |
| 067 | 38767 | 38976 | Hypothetical protein | 209/210(99%) | MN101229.1 |
| 068 | 38961 | 39803 | Hypothetical protein | 823/843(98%) | MN101229.1 |
| 069 | 39845 | 40051 | Hypothetical protein | 205/207(99%) | MK421971.1 |
| 070 | 40051 | 41523 | DNA ligase | 1428/1473(97%) | KR269720.1 |
| 071 | 41520 | 41699 | Hypothetical protein | 180/180(100%) | MH333064.1 |
| 072 | 41751 | 43877 | Hypothetical protein | 2075/2127(98%) | MG751100.1 |
| 073 | 43941 | 44219 | Hypothetical protein | 275/279(99%) | MN101226.1 |
| 074 | 44241 | 45293 | Phage baseplate tail tube cap | 1043/1053(99%) | MH333064.1 |
| 075 | 45293 | 46219 | Tail assembly protein | 919/927(99%) | KR269720.1 |
| 076 | 46229 | 46987 | Phage baseplate | 744/759(98%) | KR269720.1 |
| 077 | 46984 | 48126 | Baseplate hub subunit | 1125/1143(98%) | MN434095.1 |
| 078 | 48098 | 48625 | Phage baseplate hub | 525/528(99%) | KT239446.1 |
| 079 | 48622 | 50355 | Hypothetical protein | 1699/1734(98%) | MG751100.1 |
| 080 | 50407 | 51036 | Phage baseplate hub subunit | 629/630(99%) | KR269720.1 |
| 081 | 51033 | 51443 | Baseplate wedge subunit | 405/411(99%) | KR269720.1 |
| 082 | 51485 | 51901 | Single stranded DNA-binding protein | 415/417(99%) | MN434095.1 |
| 083 | 51932 | 52099 | Hypothetical protein | 168/168(100%) | MN434095.1 |
| 084 | 52120 | 53661 | DNA helicase | 1488/1504(99%) | MN434095.1 |
| 085 | 53658 | 53900 | DNA helicase | 242/243(99%) | MG751100.1 |
| 086 | 53959 | 54753 | Inhibitor of prohead protease | 774/795(97%) | KR269720.1 |
| 087 | 54763 | 55887 | Capsid and scaffold protein | 1073/1125(95%) | LR877331.1 |
| 088 | 55966 | 56193 | Hypothetical protein | 224/228(98%) | DQ845390.1 |
| 089 | 56193 | 56516 | Hypothetical protein | 322/324(99%) | MN101230.1 |
| 090 | 56630 | 56887 | Hypothetical protein | 254/258(98%) | MK421971.1 |
| 091 | 56898 | 57905 | RNA ligase | 984/1008(98%) | KT239446.1 |
| 092 | 57905 | 58453 | Hypothetical protein | 547/549(99%) | KR269720.1 |
| 093 | 58483 | 59763 | Capsid vertex protein | 1258/1281(98%) | MG751100.1 |
| 094 | 59845 | 61404 | Major capsid protein | 1555/1560(99%) | KR269720.1 |
| 095 | 61425 | 62237 | Phage prohead assembly protein | 807/813(99%) | MN434095.1 |
| 096 | 62270 | 63838 | Putative portal vertex protein | 1546/1569(99%) | MH333064.1 |
| 097 | 63838 | 64059 | Prohead core protein | 222/222(100%) | MN434095.1 |
| 098 | 64059 | 64484 | Capsid and scaffold protein | 425/426(99%) | MH333064.1 |
| 099 | 64484 | 65134 | Phage prohead assembly protein | 649/651(99%) | LR877331.1 |
| 100 | 65239 | 65730 | Tail fibers protein | 488/492(99%) | MN101230.1 |
| 101 | 65841 | 66473 | Hypothetical protein | 613/621(99%) | KR269720.1 |
| 102 | 66535 | 68517 | Phage tail sheath | 1909/1974(97%) | KT239446.1 |
| 103 | 68530 | 69375 | Tail sheath stabilizer and completion protein | 807/825(98%) | MN101230.1 |
| 104 | 69372 | 69881 | Phage terminase, small subunit | 498/510(98%) | KX258185.1 |
| 105 | 69859 | 71697 | Putative terminase large subunit | 1818/1839(99%) | CP062992.1 |
| 106 | 71739 | 72515 | Phage neck protein | 754/777(97%) | KX258185.1 |
| 107 | 72519 | 73460 | Phage neck protein | 912/942(97%) | KR269720.1 |
| 108 | 73517 | 75283 | Hypothetical protein | 1725/1768(98%) | KR269720.1 |
| 109 | 75292 | 75963 | Baseplate wedge subunit and tail pin | 653/672(97%) | MG751100.1 |
| 110 | 75963 | 77303 | Hypothetical protein | 1334/1341(99%) | KR269720.1 |
| 111 | 77305 | 78216 | Hypothetical protein | 900/912(99%) | KR269720.1 |
| 112 | 78216 | 80039 | baseplate wedge subunit and tail pin | 1782/1824(98%) | MN101229.1 |
| 113 | 80107 | 80610 | Hypothetical protein | 500/504(99%) | MN101229.1 |
| 114 | 80611 | 80904 | Hypothetical protein | 294/294(100%) | MN101229.1 |
| 115 | 80904 | 82871 | Baseplate wedge subunit | 1919/1968(98%) | MN101229.1 |
| 116 | 82868 | 85966 | Phage baseplate wedge initiator | 3007/3100(97%) | KR269720.1 |
| 117 | 85959 | 86984 | Baseplate wedge tail fiber connector | 1008/1026(98%) | MG751100.1 |
| 118 | 86987 | 87625 | Baseplate wedge subunit | 635/639(99%) | MH333064.1 |
| 119 | 87622 | 89352 | Phage baseplate hub structural protein/ Phage lysozyme R | 1680/1731(97%) | MG751100.1 |
| 120 | 89402 | 89851 | Head completion protein | 448/450(99%) | KY000080.1 |
| 121 | 89851 | 90678 | DNA end protector protein | 1089/1098(99%) | MH333064.1 |
| 122 | 90678 | 91280 | Phage tail completion protein | 597/603(99%) | KR269720.1 |
| 123 | 91285 | 92016 | Deoxynucleoside monophosphate kinase | 729/732(99%) | KR269720.1 |
| 124 | 92019 | 92252 | Hypothetical protein | 228/230(99%) | KY000080.1 |
| 125 | 92252 | 92707 | Hypothetical protein | 440/446(99%) | CP062992.1 |
| 126 | 92704 | 93087 | Hypothetical protein | 383/384(99%) | KY000080.1 |
| 127 | 93150 | 93329 | Hypothetical protein | 178/180(99%) | MN101230.1 |
| 128 | 93316 | 93495 | Hypothetical protein | 178/180(99%) | KR269720.1 |
| 129 | 93495 | 93680 | Hypothetical protein | 184/186(99%) | MN434095.1 |
| 130 | 93697 | 94047 | Hypothetical protein | 351/351(100%) | MN101229.1 |
| 131 | 95784 | 95936 | Hypothetical protein | 150/153(98%) | MN101229.1 |
| 132 | 95933 | 96298 | Hypothetical protein | 352/366(96%) | 352/366(96%) |
| 133 | 96285 | 96515 | Hypothetical protein | 228/231(99%) | MN434095.1 |
| 134 | 96570 | 96794 | Hypothetical protein | 222/225(99%) | MN101225.1 |
| 135 | 96966 | 97145 | Hypothetical protein | 180/180(100%) | MH333064.1 |
| 136 | 97145 | 97702 | Hypothetical protein | 548/558(98%) | MG751100.1 |
| 137 | 97703 | 98152 | Hypothetical protein | 446/450(99%) | MN101230.1 |
| 138 | 99361 | 99546 | Hypothetical protein | 186/186(100%) | MN434095.1 |
| 139 | 99682 | 99972 | Hypothetical protein | 290/291(99%) | MH729874.1 |
| 140 | 100020 | 100613 | Phage protein | 582/594(98%) | MH729874.1 |
| 141 | 100651 | 101241 | Hypothetical protein | 576/591(97%) | MN101230.1 |
| 142 | 101238 | 101600 | Phage protein | 359/363(99%) | LR877331.1 |
| 143 | 101597 | 101893 | Hypothetical protein | 286/297(96%) | LR877331.1 |
| 144 | 101890 | 102216 | Unique | 0/326（0%） | NO |
| 145 | 102216 | 102446 | Unique | 0/230（0%） | NO |
| 146 | 102446 | 102892 | Nudix hydrolase, phage-associated | 375/434(86%) | LR877331.1 |
| 147 | 102924 | 103418 | Phage lysozyme R | 487/495(98%) | LR877331.1 |
| 148 | 103419 | 103898 | Hypothetical protein | 471/479(98%) | MH333064.1 |
| 149 | 103980 | 104282 | Hypothetical protein | 302/303(99%) | MN434095.1 |
| 150 | 104372 | 104944 | Phage protein | 564/573(98%) | MN101229.1 |
| 151 | 105119 | 105652 | Phage protein | 533/534(99%) | MK421971.1 |
| 152 | 105649 | 105960 | Phage protein | 312/312(100%) | MN434095.1 |
| 153 | 106086 | 106448 | Pyruvate formate lyase | 360/363(99%) | MK421971.1 |
| 154 | 106448 | 106723 | Phage protein | 275/276(99%) | MN101225.1 |
| 155 | 106787 | 107245 | Phage endonuclease | 459/459(100%) | MH333064.1 |
| 156 | 107253 | 107798 | Hypothetical protein | 539/546(99%) | MG751100.1 |
| 157 | 107798 | 108118 | Valyl-tRNA synthetase | 319/321(99%) | KX258185.1 |
| 158 | 108115 | 108507 | Hypothetical protein | 390/393(99%) | KR269720.1 |
| 159 | 108516 | 108983 | Hypothetical protein | 462/468(99%) | KR269720.1 |
| 160 | 109008 | 109175 | Phage protein | 168/168(100%) | KR269720.1 |
| 161 | 109175 | 109774 | Thymidine kinase | 590/600(98%) | KR269720.1 |
| 162 | 109752 | 109949 | Hypothetical protein | 195/198(98%) | KR269720.1 |
| 163 | 110036 | 110170 | Hypothetical protein | 135/135(100%) | KR269720.1 |
| 164 | 110234 | 110617 | Hypothetical protein | 382/384(99%) | CP062992.1 |
| 165 | 110664 | 110876 | Phage protein | 213/213(100%) | MN101230.1 |
| 166 | 110888 | 111175 | rI lysis inhibition regulator membrane protein | 288/288(100%) | MN101226.1 |
| 167 | 111162 | 111548 | Phage protein | 385/387(99%) | MN101230.1 |
| 168 | 111653 | 112141 | Hypothetical protein | 468/489(96%) | MN101225.1 |
| 169 | 112233 | 112499 | Hypothetical protein | 267/267(100%) | KX258185.1 |
| 170 | 112502 | 112693 | Hypothetical protein | 192/192(100%) | KR269720.1 |
| 171 | 112683 | 112907 | Hypothetical protein | 223/225(99%) | KR269720.1 |
| 172 | 112910 | 113401 | Hypothetical protein | 467/483(97%) | KX258185.1 |
| 173 | 113398 | 113979 | Hypothetical protein | 573/582(98%) | KT239446.1 |
| 174 | 113969 | 114190 | Hypothetical protein | 205/220(93%) | LR877331.1 |
| 175 | 114172 | 114594 | Unique | 0/422（0%） | NO |
| 176 | 114591 | 115601 | Hypothetical protein | 937/1013(92%) | MN101230.1 |
| 177 | 115657 | 115794 | Hypothetical protein | 137/138(99%) | MN434095.1 |
| 178 | 115815 | 116030 | Hypothetical protein | 213/216(99%) | MN101229.1 |
| 179 | 116147 | 116362 | Hypothetical protein | 213/216(99%) | MN101229.1 |
| 180 | 116440 | 117417 | Thioredoxin | 956/978(98%) | MH729874.1 |
| 181 | 117460 | 117606 | Hypothetical protein | 146/147(99%) | MK421971.1 |
| 182 | 117603 | 118028 | Hypothetical protein | 415/426(97%) | MH333064.1 |
| 183 | 118025 | 118360 | Hypothetical protein | 335/336(99%) | MH333064.1 |
| 184 | 118357 | 118656 | Hypothetical protein | 300/300(100%) | MH333064.1 |
| 185 | 118658 | 118846 | Hypothetical protein | 188/189(99%) | MH333064.1 |
| 186 | 118843 | 119130 | Hypothetical protein | 280/288(97%) | LR877331.1 |
| 187 | 119211 | 119375 | Hypothetical protein | 164/165(99%) | MK421971.1 |
| 188 | 119372 | 119638 | Hypothetical protein | 258/267(97%) | MH333064.1 |
| 189 | 119696 | 120484 | Hypothetical protein | 784/788(99%) | KT239446.1 |
| 190 | 120551 | 121573 | Unique | 0/1022（0%） | NO |
| 191 | 121570 | 121794 | Hypothetical protein | 144/169(85%) | MN434095.1 |
| 192 | 121791 | 122105 | Hypothetical protein | 281/316(89%) | MG751100.1 |
| 193 | 122102 | 122437 | Hypothetical protein | 327/336(97%) | MN101229.1 |
| 194 | 122434 | 122946 | Hypothetical protein | 508/513(99%) | MN101230.1 |
| 195 | 122939 | 123112 | Hypothetical protein | 174/174(100%) | MN101230.1 |
| 196 | 123102 | 123392 | Hypothetical protein | 280/291(96%) | MK421971.1 |
| 197 | 123355 | 123624 | Thioredoxin | 265/270(98%) | MN101229.1 |
| 198 | 123617 | 123805 | Hypothetical protein | 187/189(99%) | MN101230.1 |
| 199 | 123824 | 123994 | Hypothetical protein | 171/171(100%) | MN434095.1 |
| 200 | 123996 | 124385 | Hypothetical protein | 383/390(98%) | MH333064.1 |
| 201 | 124364 | 124522 | Hypothetical protein | 159/159(100%) | MH333064.1 |
| 202 | 124506 | 124958 | Hypothetical protein | 448/453(99%) | MN434095.1 |
| 203 | 124951 | 125259 | Hypothetical protein | 304/309(98%) | MG751100.1 |
| 204 | 125296 | 125769 | Recombinase endonuclease VII | 474/474(100%) | MH333064.1 |
| 205 | 125766 | 127604 | Ribonucleotide reductase of class III (anaerobic), large subunit | 1804/1839(98%) | MH729874.1 |
| 206 | 127576 | 127809 | Hypothetical protein | 234/234(100%) | MH729874.1 |
| 207 | 127781 | 128251 | Ribonucleotide reductase of class III (anaerobic), activating protein | 465/471(99%) | MH729874.1 |
| 208 | 128262 | 128360 | Hypothetical protein | 99/99(100%) | MH729874.1 |
| 209 | 128369 | 128581 | Hypothetical protein | 213/213(100%) | KR269720.1 |
| 210 | 128578 | 128880 | Glutaredoxin | 303/303(100%) | MN101229.1 |
| 211 | 129066 | 129254 | Hypothetical protein | 186/186(100%) | MH729874.1 |
| 212 | 129251 | 129493 | Hypothetical protein | 243/243(100%) | MH729874.1 |
| 213 | 129558 | 129890 | Hypothetical protein | 333/333(100%) | MH729874.1 |
| 214 | 129887 | 130144 | Hypothetical protein | 258/258(100%) | MH729874.1 |
| 215 | 130137 | 130421 | Hypothetical protein | 285/285(100%) | MH729874.1 |
| 216 | 130414 | 130638 | Hypothetical protein | 223/225(99%) | MN434095.1 |
| 217 | 130626 | 130904 | Hypothetical protein | 279/279(100%) | MN434095.1 |
| 218 | 130901 | 131146 | Hypothetical protein | 246/246(100%) | KR269720.1 |
| 219 | 131213 | 131761 | Sigma factor | 549/549(100%) | MN101229.1 |
| 220 | 131745 | 131975 | Hypothetical protein | 231/231(100%) | MN434095.1 |
| 221 | 131977 | 132279 | Hypothetical protein | 303/303(100%) | MN434095.1 |
| 222 | 132257 | 132454 | Hypothetical protein | 198/198(100%) | MH729874.1 |
| 223 | 132540 | 132785 | Hypothetical protein | 246/246(100%) | MN434095.1 |
| 224 | 132855 | 134051 | Hypothetical protein | 1155/1197(96%) | KT239446.1 |
| 225 | 134121 | 135143 | Phage recombination-related endonuclease | 1022/1023(99%) | MN101230.1 |
| 226 | 135140 | 136822 | Phage recombination-related endonuclease | 1637/1683(97%) | LR877331.1 |
| 227 | 136819 | 137013 | Hypothetical protein | 195/195(100%) | KY000080.1 |
| 228 | 137024 | 137401 | RNA polymerase binding protein | 378/378(100%) | MK421971.1 |
| 229 | 137437 | 138138 | Sliding clamp DNA polymerase accessory protein | 695/702(99%) | MN101226.1 |
| 230 | 138187 | 139149 | Replication factor C small subunit/Phage DNA polymerase clamp loader subunit | 949/963(99%) | KR269720.1 |
| 231 | 139146 | 139715 | Phage DNA polymerase clamp loader subunit | 556/570(98%) | KX258185.1 |
| 232 | 139718 | 140080 | Translational repressor protein | 354/363(98%) | KR269720.1 |
| 233 | 140080 | 140280 | Hypothetical protein | 201/201(100%) | MG751100.1 |
| 234 | 140373 | 143081 | Putative DNA polymerase | 2648/2709(98%) | KX258185.1 |
| 235 | 143150 | 143593 | Hypothetical protein | 433/444(98%) | MK421971.1 |
| 236 | 143604 | 143810 | Hypothetical protein | 206/207(99%) | MK421971.1 |
| 237 | 143819 | 144553 | Hypothetical protein | 718/735(98%) | MH333064.1 |
| 238 | 144550 | 145386 | Glucosyl transferase | 814/837(97%) | MN434095.1 |
| 239 | 145413 | 145634 | Hypothetical protein | 222/222(100%) | MG751100.1 |
| 240 | 145717 | 146892 | Recombination protein | 1167/1176(99%) | MN101229.1 |
| 241 | 146882 | 147232 | Capsid and scaffold protein | 351/351(100%) | MN101226.1 |
| 242 | 147242 | 148681 | Phage DNA primase/helicase | 1421/1440(99%) | MN101229.1 |
| 243 | 148691 | 148924 | Hypothetical protein | 233/234(99%) | MN434095.1 |
| 244 | 148990 | 149283 | Hypothetical protein | 288/294(98%) | KR269720.1 |
| 245 | 149280 | 149489 | Hypothetical protein | 210/210(100%) | KT239446.1 |
| 246 | 149505 | 149669 | Hypothetical protein | 165/165(100%) | KR269720.1 |
| 247 | 149708 | 150730 | DNA primase | 1007/1022(99%) | KR269720.1 |
| 248 | 150750 | 150941 | Hypothetical protein | 191/192(99%) | MG751100.1 |
| 249 | 151008 | 151529 | dCTPase | 513/522(98%) | KT239446.1 |
| 250 | 151529 | 151666 | Hypothetical protein | 138/138(100%) | MN101230.1 |
| 251 | 151696 | 151932 | Hypothetical protein | 231/237(97%) | KX258185.1 |
| 252 | 152011 | 152202 | Hypothetical protein | 192/192(100%) | MN434095.1 |
| 253 | 152180 | 152689 | Hypothetical protein | 504/510(99%) | LR877331.1 |
| 254 | 152689 | 152877 | Hypothetical protein | 189/189(100%) | MN434095.1 |
| 255 | 152948 | 153637 | Hypothetical protein | 682/690(99%) | MG751100.1 |
| 256 | 153718 | 154473 | Putative anti-sigma factor | 734/756(97%) | KX258185.1 |
| 257 | 154473 | 154787 | Hypothetical protein | 312/315(99%) | MN101226.1 |
| 258 | 154784 | 156115 | DNA helicase | 1309/1332(98%) | MN101229.1 |
| 259 | 156122 | 156376 | Hypothetical protein | 255/255(100%) | MN101229.1 |
| 260 | 156369 | 157058 | Exonuclease | 661/690(96%) | MN101226.1 |
| 261 | 157058 | 157225 | Hypothetical protein | 165/168(98%) | KR269720.1 |
| 262 | 157289 | 157729 | Hypothetical protein | 425/441(96%) | KT239446.1 |
| 263 | 157794 | 158009 | Hypothetical protein | 213/216(99%) | MH333064.1 |
| 264 | 158009 | 158374 | Hypothetical protein | 358/366(98%) | MG751100.1 |
| 265 | 158368 | 158703 | Hypothetical protein | 332/336(99%) | MK421971.1 |
| 266 | 158690 | 158866 | Hypothetical protein | 169/177(95%) | MK421971.1 |
| 267 | 158866 | 158955 | Hypothetical protein | 90/90(100%) | MN434095.1 |
| 268 | 158952 | 159200 | Hypothetical protein | 242/249(97%) | MH333064.1 |
| 269 | 159279 | 159647 | Hypothetical protein | 310/358(87%) | KR269720.1 |
| 270 | 159678 | 161516 | Phage DNA topoisomerase large subunit | 1760/1839(96%) | MH729874.1 |
| 271 | 161557 | 161709 | Hypothetical protein | 150/153(98%) | MN434095.1 |
| 272 | 161849 | 162055 | Hypothetical protein | 207/207(100%) | MN101230.1 |
| 273 | 162066 | 164234 | Hypothetical protein | 2100/2169(97%) | CP062992.1 |
| 274 | 164235 | 165146 | Hypothetical protein | 902/912(99%) | MN101230.1 |
| 275 | 165189 | 165485 | Hypothetical protein | 291/297(98%) | LR877331.1 |
